# The Role of Autophagy in Lupus Nephritis

**DOI:** 10.3390/ijms161025154

**Published:** 2015-10-22

**Authors:** Linlin Wang, Helen Ka Wai Law

**Affiliations:** Department of Health Technology and Informatics, Faculty of Health and Social Sciences, The Hong Kong Polytechnic University, Hunghom, Hong Kong, China; E-Mail: 13903160r@connect.polyu.hk

**Keywords:** autophagy, lupus nephritis, systemic lupus erythematosus

## Abstract

Systemic lupus erythematosus (SLE) is a multifactorial autoimmune disease characterized by the generation of immune responses to self-antigens. Lupus nephritis is one of the most common and severe complications in SLE patients. Though the pathogenesis of lupus nephritis has been studied extensively, unresolved questions are still left and new therapeutic methods are needed for disease control. Autophagy is a conserved catabolic process through which cytoplasmic constituents can be degraded in lysosome and reused. Autophagy plays vital roles in maintaining cell homeostasis and is involved in the pathogenesis of many diseases. In particular, autophagy can affect almost all parts of the immune system and is involved in autoimmune diseases. Based on genetic analysis, cell biology, and mechanism studies of the classic and innovative therapeutic drugs, there are growing lines of evidence suggesting the relationship between autophagy and lupus nephritis. In the present review, we summarize the recent publications investigating the relationship between autophagy and lupus nephritis and provide a new perspective towards the pathogenesis of lupus nephritis.

## 1. Introduction

Systemic lupus erythematosus (lupus; SLE) is a prototype of chronic autoimmune inflammatory disease characterized by loss of tolerance against self-antigens, polyclonal autoantibody production, immune complex formation, and deposition in different part of the body, leading to detrimental inflammation and multi-organ injuries [1]. Among the wide spectrum of complications of SLE, one of the most common and severe is lupus nephritis. Lupus nephritis occurs in 50%–70% of SLE patients in the first five years of diagnosis [2]. It may lead to permanent renal damage and chronic kidney disease. Renal involvement early in the course of SLE becomes a major predictor of poor prognosis [2]. Although the disease has been known for centuries, the pathogenesis remains unclear. The current treatments for lupus nephritis rely mainly on glucocorticoids and immunosuppressants. They are partially effective and have considerable adverse effects, especially for long-time use. New therapeutic strategies and drugs are eagerly demanded.

Autophagy is an evolutionarily conserved catabolic process to degrade cytoplasmic contents through lysosomes [3]. Depending on the ways delivering cytoplasmic materials to the lysosomes, at least three major types of autophagy have been identified: macroautophagy, microautophagy, and chaperone-mediated autophagy (CMA), with macroautophagy being the best understood [4]. Macroautophagy (hereafter called autophagy) describes a degradation process, through which unique double-membrane vesicles (autophagosomes) are formed to sequester a targeted portion of cytoplasm, then fuse with lysosomes to form autolysosomes and degrade the internal materials. Microautophagy involves direct engulfment of cytoplasmic cargo at the lysosomal surface [5]. Both macroautophagy and microautophagy have the ability to engulf structures through non-selective and selective mechanisms [6]. CMA involves direct translocation of unfolded proteins across the lysosome membrane with the help of chaperones. Particularly, cytosolic proteins containing a pentapeptide motif will be recognized by chaperone heat shock cognate 70 (Hsc70) and delivered to the surface of lysosomal membrane through the interaction between Hsc70 and the receptor lysosome-associated membrane protein 2A (LAMP-2A) [6,7].

Autophagy exists at basal level in all cell types and will be activated in response to different stresses [8]. Autophagy is being regulated precisely and plays a vital role in maintaining cell homeostasis. It is a typical protective, pro-survival response at the initiation of damages. However, if hyperactivated, it will ultimately kill the cells, known as type II programmed cell death. Autophagy has many physiological roles and is involved in the pathogenesis of diverse diseases, including cardiovascular diseases, neuro-degeneration, obesity, aging, cancers, infections, autoinflammatory diseases, and autoimmune diseases (reviewed in [9,10,11]).

Especially, autophagy also affects diverse aspects of immune system (reviewed in [12,13]). Autophagy can directly eliminate intracellular pathogens (xenophagy [14]). Autophagy participates in inflammation. It can regulate type I interferon production by virally-infected cells, however some reports are controversial [15,16,17,18]. Inflammasomes are molecular platforms activated under infection or stresses which trigger the maturation of proinflammatory cytokines and play critical roles in host defence and inflammation. Evidence shows that autophagy can negatively regulate inflammasome activation [19]. Assembled inflammasomes would recruit autophagic adaptor p62 to assist their delivery to autophagosomes and the degradation within them [20]. Autophagy can promote MHC class II presentation of endogenous antigens [21]. Autophagy is vital for T cell and B cell homeostasis [22]. Besides, autophagy is involved in autoimmunity. Genome-wide association studies (GWAS) have linked polymorphisms in autophagy-related genes to some autoinflammatory and autoimmune diseases, specifically in inflammatory bowel disease (IBD) and SLE [23]. As lupus nephritis results from immune system disorders, we hypothesize that autophagy may be involved in lupus development and renal injuries. In this review, we discuss recent publications on the relationship between autophagy and lupus nephritis, with a view of providing new insights and research directions into the pathogenesis and treatment of lupus nephritis.

## 2. Genes

Lupus is a complex disease and many factors interplay and contribute to the disease, including the genetic, environmental, hormonal and immunoregulatory factors. The rapid development of GWAS has revealed more than 40 genes associated with SLE (based on the data from the National Human Genome Research Institute’s Catalog of Published Genome-Wide Association Studies, http://www.ebi.ac.uk/gwas, [24]). Among them, some genes have shown to be highly related to autophagy, including *ATG5* [25,26], *ATG7* [27], *IRGM* (immunity-related GTPase family M) [27], *DRAM1* (DNA damage regulated autophagy modulator) [28], *CDKN1B* (cyclin-dependent kinase inhibitor 1 B) [28], *APOL1* (apolipoprotein L1) [29], and *MTMR3* (Myotubularin-related phosphatase 3) [30]. We will point out some genes which are highly related to lupus nephritis.

### 2.1. APOL1

Apolipoprotein L1 (APOL1) is a BH3-only protein that can induce autophagic cell death in a variety of cells when it is overexpressed [31]. In 2013, Larsen and colleagues examined 546 renal biopsies from African American SLE patients and 26 cases of collapsing glomerulopathy (CG) were identified. DNA was extracted from the biopsy tissues and genotyped for *APOL1* risk alleles. Their results showed a strong association between *APOL1* and SLE-associated CG (*p* < 0.001). To explain the role of *APOL1* in the pathogenesis of SLE-associated CG, they described a “two-hit hypothesis”, in which presence of *APOL1* risk alleles together with altered inflammatory milieu helped to disease development [32]. In a subsequent study, Freedman and colleagues genotyped the SNPs in *APOL1* (rs73885319 and rs60910145 in G1 allele, and rs71785313 in G2 allele) in 855 African American SLE patients with end-stage renal disease (ESRD) and 534 African American SLE patients without renal involvement. Their results also revealed a strong association between *APOL1* G1/G2 alleles and the risk of SLE-ESRD in African Americans (odds ratio 2.57, recessive model *p* = 1.49 × 10^−9^) [29]. In a more recent focal segmental glomerulosclerosis clinical trial, Kopp *et al.* [33] studied the *APOL1*-asosociated nephropathy and *APOL1* renal risk allele frequency in 94 cases of children and young adults. It was demonstrated that *APOL1* risk alleles is associated with more severe kidney fibrosis at diagnosis and more rapid progression into ESRD. As elaborated in the Editorial comments by Larsen and Freedman [34], the pathologic mechanisms and the primary kidney cell types related to *APOL1* remain unknown. Combining the biochemistry and clinical data, it is probable that the expression of APOL1 is associated with autophagy in renal tissues of SLE patients. Further investigation is needed to substantiate this hypothesis.

### 2.2. MTMR3

Myotubularin-related phosphatase 3 (MTMR3) is one of the phosphatidylinositol 3-phosphate (PI3P) phosphatases [35]. These phosphatases together with PI kinases reciprocally regulate the cellular level of PI3P and are involved in constitutive autophagy initiation and autophagosome size [36]. Zhou and colleagues [30] identified a significant association between a genetic variant of *MTMR3* (rs9983A) and the risk of lupus nephritis in northern Han Chinese populations. Expression analysis revealed lower *MTMR3* transcriptions in the blood samples with this variant and in renal biopsy samples from lupus nephritis patients.

## 3. Environmental Risk Factors

Epidemiologic studies reveal the importance of environmental factors in lupus and lupus nephritis [37]. A genetically susceptible individual exposed to risk environments, such as ultraviolet (UV) light [38], Epstein-Barr virus (EBV) infections [39,40], and smoking [41], will facilitate and enhance the disorders of immune systems and aggravate diseases.

### 3.1. UV Light

UV light exposure is a risk factor for lupus development. Photosensitivity is a well-known characteristic of lupus and there are reports that sunlight can lead to cutaneous damage and life-threatening lupus nephritis [42]. Kemp and colleagues [43] exposed keratinocytes, monocytes and T cells to UV light and demonstrated that UV-induced DNA damage led to lower protein expressions of AMBRA1 and ULK1, which negatively regulated STING (stimulator of interferon genes) and further led to activation of transcription factor IRF3 (interferon regulatory factor 3). Although the authors did not evaluate the autophagy process in their cells after UV exposure, AMBRA1 and ULK1 are important machineries in autophagy and these results warrant for further investigations on the relationship among UV exposure, dysregulation of autophagy, and organ damage in lupus patients.

### 3.2. Epstein-Barr Virus (EBV)

The association between EBV infection and lupus development has been investigated for decades [44]. Nearly 100% of lupus patients are positive for EBV-VCA (EBV viral capsid antigen) IgG response [45,46]. Some antibodies against EBV antigens such as EBNA-1 (EB nuclear antigen 1) and EBNA-2 (EB nuclear antigen 2), can cross-react with autoantigenic proteins such as Sm and Ro and support the hypothesis that some humoral autoimmunity in lupus arises through molecular mimicry [47]. Besides, increased EBV load, altered cellular and humoral immune response to EBV, and increased viral gene expressions in peripheral blood mononuclear cells in lupus patients, implicated the important roles of EBV in lupus pathogenesis [44].

The roles of EBV infections in lupus nephritis have also been investigated. Yu and colleagues [48] collected 58 renal tissue samples from patients with lupus nephritis and compared them with samples from patients with non-glomerular hematuria and patients with minimal change nephropathy. They detected expression of EBV-latent membrane protein 1 (LMP1) in renal tissues using immunohistochemistry while they detected the expression of EBV-encoded RNA 1 (EBER-1) using *in situ* hybridization. Results showed that positive rates of renal viral proteins EBER-1 and LMP-1 were significantly higher in lupus patients than those in control groups. And the percentage of anti-Sm antibody positive cases in lupus patients was also higher in renal EBV-positive groups than EBV-negative groups. Ding and colleagues [49] examined 51 renal samples from young patients with lupus nephritis (aged from 6 to 16 years old) and found similar results. Positive rate of LMP1 in renal tissues was higher in young patients with lupus nephritis than that in control group and the proportion of young patients positive for anti-Sm antibody was higher in LMP1 positive group. These results suggested that renal EBV infection might be relevant to renal injuries in patients with lupus nephritis.

Autophagy is involved in EBV infections. LMP1 could induce autophagy in B cells in a dose-dependent manner. Inhibiting autophagy promoted accumulation of LMP1 in EBV-positive cells [50]. Autophagy is also involved in MHC class II mediated presentation of endogenous antigen EBNA-1 to CD4^+^ T cells and participated in antiviral responses [51]. Thus, autophagy may affect disease course partially though dysregulated immunity linked to EBV.

## 4. Cells

Lupus is a systemic autoimmune disorder. Many cells in innate and adaptive immunity are affected and dysregulated, especially including the B cells, T cells, dendritic cells (DCs) and phagocytes. For example, Clarke *et al.* [52] demonstrated that autophagy in splenic CD19^+^ B cells from NZB/W lupus mice was increased compared with C57BL/6 control mice. Increased autophagy was also observed in B cells in human lupus patients. Differentiation from B cells to plasma cells also required autophagy. Inhibiting autophagy by using Atg7 deficient B cells reduced the number and viability of CD19^+^CD138^+^ plasma cells and decreased secretion of IgM. Chemical autophagy inhibitor 3-MA can also dramatically reduced plasmablast formation and cell proliferation. Previous studies have investigated the role of autophagy in plasma cells differentiation and homeostasis [53]. By using CD19-cre×Atg5 ^flox/flox^ mice in which Atg5 was conditionally deleted in CD19-expression cells, Pengo and colleagues [53] revealed that autophagy showed a negative control on endoplasmic reticulum (ER) expansion and immunoglobulin synthesis in plasma cells, via a crucial transcriptional repressor Blimp-1, and demonstrated a protective role for plasma cell maintenance by sustaining energy metabolism and cell viability. Conway and colleagues [54] also demonstrated the importance of autophagy related protein Atg5 in late B cell activation and plasma cell differentiation. Gros *et al.* [55] reported that central and peripheral T cells from lupus-prone mice (MRL/lpr and NZB/W mice) exhibited increased autophagic flux and autophagosomes compared with CBA/J and BALB/c control mice. Peripheral T cell from lupus patients also exhibited increased number of autophagic vacuoles.

Besides, lupus can lead to multiple end-organ injuries, among which renal involvement is associated with more severe symptoms and poorer prognosis. Thus renal parenchyma cell injuries should also be considered. Here we will discuss different types of cells which are involved in renal damage and present dysregulated autophagy.

### 4.1. Neutrophils and Neutrophil Extracellular Trap (NET)

Neutrophils are involved in the pathogenesis of lupus. Genomic studies showed that neutrophil-specific genes are the second most prevalent peripheral-blood mononuclear cell transcriptional signature in children with SLE [24,56]. Infections can initiate flares and are major causes of mortality in SLE patients. When facing infections, neutrophils are recruited to infection sites and release NETs to play the antimicrobial role. The NETs are composed of decondensed chromatin fibers and neutrophil proteins, which are released into the extracellular matrix as the neutrophil undergoes a unique form of cell death—NETosis, to trap and kill invading pathogens. The NETs can activate plasmacytoid dendritic cells through Toll Like Receptors (TLRs) to produce massive type I interferon. If the released NETs cannot be removed timely, they may become a source to be presented as self-antigens and stimulate more autoantibody production. Hakkim *et al.* [57] demonstrated that impairments of NET degradation existed in a subset of SLE patients (36.1% of the 61 SLE patients) and the impairments are correlated with lupus nephritis. Serum DNase 1 is essential for degrading NETs. Existence of DNase 1 inhibitors in serum or anti-NET antibody that prevented DNase 1 accessing to NETs may explain the inhibitory mechanisms of NETs degradation. SLE patients who have impaired NETs degradation were more likely to develop lupus nephritis. They also had higher titers of anti-ds DNA antibodies (which are correlated with renal disease) than the degraders. Martinez-Valle *et al.* reported that DNase 1 activities in SLE patients were significantly lower than that in control group (13.69 ± 8.52 *vs.* 24.75 ± 12.32 μg/mL, *p* < 0.005) [58]. Leffler *et al.* found that NETs could activate complement *in vitro* and deposited C1q could inhibit NET degradation by direct inhibition of DNase 1 [59].

Evidence shows that autophagy affects the formation and release of NET. Itakura *et al.* [60] analysed the kinetics of NET release by monitoring the extracellular release of DNA and one well-known NET protein neutrophil elastase (NE) from activated neutrophils, using the cell-impermeable DNA dye Sytox green and immunofluorescence staining of NE protein. Bacteria-derived peptide formyl-Met-Leu-Phe (fMLP) can activate and stimulate neutrophils to lease NET, proved by increased percentage of Sytox-positive cells, in a time-dependent manner. After pretreatment with mTOR inhibitors rapamycin or WYE-354 (which can induce autophagy), more Sytox-positive neutrophils and increased NE expression were detected under fMLP stimulation, compared with treatment with vehicle or fMLP alone. Meanwhile, mTOR inhibitors rapamycin and WYE-354 can promote autophagosome formation and induce autophagy. These results indicated that suppressed mTOR activity and increased autophagy may lead to increased NET release, and thus may affect lupus development.

### 4.2. Macrophages

The macrophage is an important component of the innate immune system, participating in antigen presentation, removal of dying cells and their released components, cytokines production and many other immunological responses. In lupus patients, deficient phagocytic capacity of macrophages was observed and led to accumulation of cell materials, which may provide a source of autoantibodies [61]. Evidences showed that autophagy could regulate macrophage functions and cell homeostasis and participated in the pathogenesis of lupus nephritis. Li and colleagues [62] detected significantly increased LC3-II expression in spleen and kidney macrophages from activated lymphocytes-derived DNA (ALD-DNA) induced lupus mice when compared with those in control mice. For *in vitro* experiments, ALD-DNA stimulation can induce autophagy in cultured RAW264.7 macrophages, proved by increased expressions of LC3-II, beclin 1 and Atg5. The authors further generated Beclin 1 knockdown macrophages by using a lentiviral shRNA. Then they adoptively transferred these autophagy-suppressed macrophages into lupus mice which were previously depleted of the original macrophages with dichloromethylenediphosphonic acid disodium salts (DMDP). Results showed alleviated symptoms in lupus mice receiving these autophagy-suppressed macrophages, demonstrated by relieved renal pathological severity, decreased anti-dsDNA titer, and declined urine protein. These results suggested that increased autophagy in macrophage may play an important role in the pathogenesis of murine lupus and kidney damage.

### 4.3. Podocytes

Podocyte, also called visceral glomerular epithelial cells, is one of the three components forming the glomerular filtration barrier. They are injured in many forms of glomerular diseases, including lupus nephritis. Cumulative evidence has revealed protective roles of autophagy in podocytes injured by chemicals [63], aging [64] or diseases [65]. Especially, Hartleben *et al.* [64] generated podocyte-specific Atg5 knockout mice (Atg5^Δpodocyte^) to indicate the protective roles of autophagy in podocyte homeostasis and glomerular diseases susceptibility. Atg5^Δpodocyte^ mice developed proteinuria and glomerulosclerosis after 20 to 24 month follow-up compared with controls. Enhanced ER stress and accumulated aggregates of ubiquitinated proteins led to loss of podocytes. Besides, they also demonstrated that glomerular diseases also led to upregulation of autophagy in podocytes. When inducing glomerular disease models in asymptomatic young Atg5^Δpodocyte^ mice, they observed tremendous increase of albuminuria in autophagy-deficient mice compared to the controls. These results implied the important roles of autophagy in podocyte survival.

However, there are less direct evidences showing the role of autophagy in podocyte injuries in lupus nephritis. A German research group observed that podocytes could uptake SLE autoantibodies. When stimulated by anti-dsDNA autoantibodies which were isolated from lupus patients, podocytes took up and aggregated these antibodies in cytosolic speckles. These aggregates harmed podocytes survivals and could be degraded by autophagy. After inhibition of autophagy with Bafilomycin A or MG132, the number of cytoplasmic aggregates significantly increased [66]. These results indicate that autophagy participates in autoantibodies degradation within podocytes and is involved in regulating podocytes injuries. Maintaining podocyte health may be new therapeutic strategies for lupus nephritis.

## 5. Drugs

The current treatments for lupus nephritis are mainly dependent on glucocorticoids and immunosuppressants [67]. They affect disease process through different mechanisms and the specific mechanisms within individual diseases are still unrevealed. These medicines are partially effective and have considerable adverse effects, especially for long-time use. Biologic agents with more specific targets represent a promising direction of treatments but clinical trials and extensive follow-ups are needed [67].

Many drugs for lupus can act as autophagy regulators, however their effects appear paradoxical on autophagy process. Glucocorticoid is a classical drug used for lupus for decades and has wide suppressant effects of the whole immune system. Recent evidences show that corticosteroid can inhibit cytoplasmic calcium signaling and induce autophagy in WEHI 7.2 cells (a CD4/CD8 double positive T cell lines) [68]. Chloroquine (CQ) or hydroxychloroquine (HCQ), which are first regarded as antimalarial agents, also present numerous immunomodulatory effects and belong to the first-line therapies for lupus. However, they belong to autophagy inhibitors, as they can raise the lysosomal pH value and impaired the last step of autophagy and autophagic protein degradation. Besides acting as autophagy inhibitor, CQ/HCQ can reduce proinflammatory cytokines production, inhibit BCR/TCR-mediated calcium signaling, absorb and block UV reaction, and inhibit TLR signalings, all of which may contribute to their beneficial effects in lupus patients [69].

### 5.1. Inhibition of Activated mTOR Pathway in Lupus Nephritis

Activated mTOR pathway has been detected in lupus nephritis and has emerged as a central pathway for the pathogenesis [70]. Treatments with rapamycin and other mTOR inhibitors, have displayed protective and benefit effects on murine lupus nephritis [71,72] and patients with lupus nephritis [73]. A Greek research group studied 32 female NZBW/F1 mice divided into four groups: (1) healthy control group (HG), in which mice were sacrificed before proteinuria development; (2) untreated group (UG), in which the mice were left untreated and developed into lupus nephritis; (3) preventive group (PG), in which mice were treated with rapamycin before they developed proteinuria; and (4) therapeutic group (TG), in which rapamycin was given after the mice were presented with severe proteinuria. Results showed that total and phosphorylated form of Akt and mTOR were increased in the kidney cortex in mice with lupus nephritis when compared with HG mice. Rapamycin intervention (in PG and TG) prolonged mice survival, reduced proteinuria, serum creatinine and anti-ds DNA titer, and alleviated renal histological lesions. They also proved that rapamycin intervention reduced the Akt and mTOR expression in TG and PG mice. These results indicated abnormal activated PI3K/Akt/mTOR pathway in glomerulus in murine lupus nephritis [74]. As this pathway is one of the most important strategies to regulate autophagy, this result implies a relationship between autophagy and lupus nephritis.

Moreover, rapamycin could act as immunosuppressant, which will also benefit patients with lupus nephritis. The effects of rapamycin on lupus nephritis and the underlying mechanisms are complex. Besides its benefits, rapamycin nephrotoxicity is observed to develop in clinical practice [75]. Different dose of rapamycin also exhibit different effects. Zhang and his colleague observed that low dose of rapamycin increased the frequency of antigen-active lymphocytes and decreased activation-induced cell death in T cells, resulting in exacerbated autoimmune experimental uveitis in B10.RIII mice [76]. Thus, we need to carefully differentiate its effects in specific situations.

### 5.2. P140

P140 is a splicesomal peptide (sequence 131–151 of the U1-70K small nuclear ribonucleoprotein) phosphorylated at Ser140. In a Phase IIb clinical trial, P140 (Lupuzor) treatment improved Systemic Lupus Erythematosus Disease Activity Index (SLEDAI) score in lupus patients [77]. Administration of P140 in saline to MRL/lpr lupus-prone mice before they presented symptoms could prolong mice survival, reduce proteinuria, and decrease titer of anti-dsDNA antibodies [78]. P140 treatment could also alleviate renal vasculitis, glomerulonephritis and dermatitis in MRL/lpr mice. Evidences showed that P140 could bind with chaperone HSC70 protein (which played a vital role in CMA), decrease its expression in MRL/lpr splenic B cells and impaired the refolding properties of this chaperone. P140 treatment could suppress the autophagic flux, with increased accumulation of p62 and LC3-II in B cells from MRL/lpr mice [79]. The effects of P140 on lupus nephritis indicated the potential role of autophagy on this disease.

## 6. Conclusions

**Figure 1:**
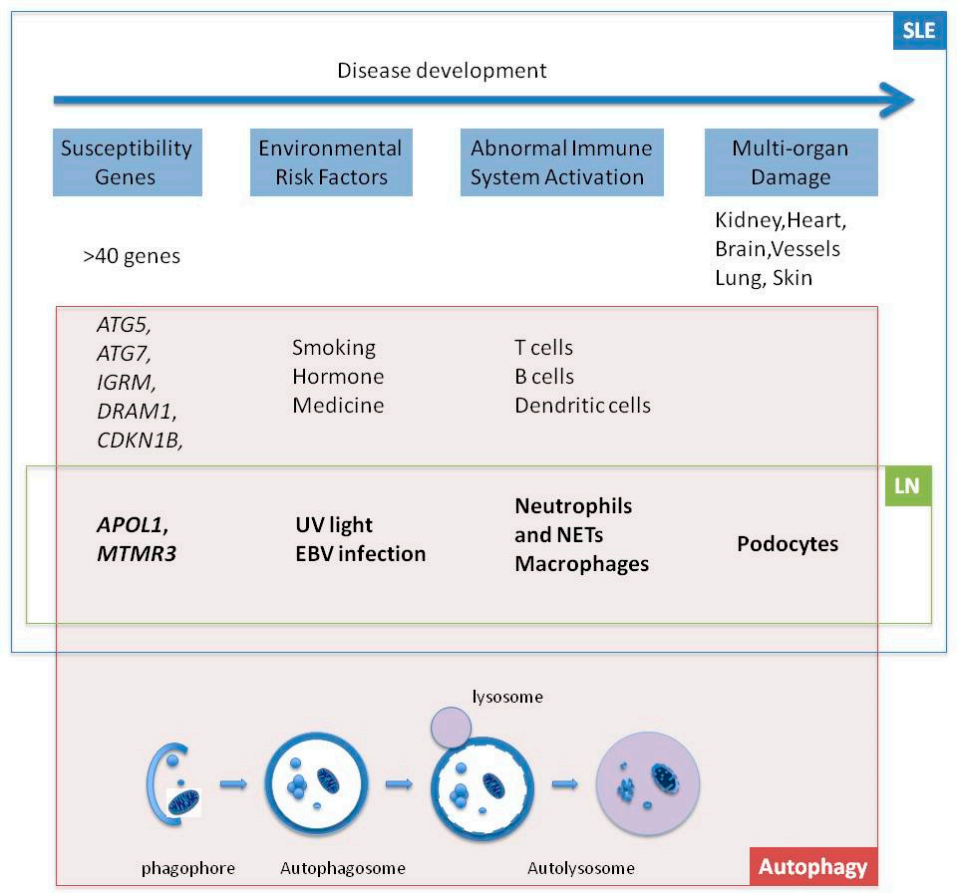
The roles of autophagy in lupus nephritis. Abbreviations: SLE: systemic lupus erythematosus; LN: lupus nephritis; EBV: Epstein–Barr virus; NETs: neutrophil extracellular traps.

There are emerging evidences indicating the relationship between autophagy and lupus nephritis. GWAS in human studies reveals autophagy-relative genes associated with lupus nephritis. Environmental risk factors for lupus nephritis can regulate the autophagy process. Autophagy can affect functions of diverse immune cells and the immune responses. The widely-used first-line drugs and innovative drugs for treatment of lupus nephritis can function as autophagy regulators. The current roles of autophagy in lupus nephritis are summarized in Figure 1. Further understanding the roles of autophagy in lupus nephritis and other autoimmune diseases, may develop new views to interpret the mechanisms of these complex diseases and autophagy process may provide new targets for diagnosis and therapy of autoimmune diseases.
